# Lymph Node Yield and Lymph Node Ratio for Prognosis of Long-Term Survival in Gastric Carcinoma

**DOI:** 10.3390/cancers17030414

**Published:** 2025-01-27

**Authors:** Olof Jannasch, Martin Schwanz, Ronny Otto, Michal Mik, Hans Lippert, Pawel Mroczkowski

**Affiliations:** 1 Department for Visceral Vascular and Emergency Surgery and VIGO, Municipial Hospital Magdeburg, 39130 Magdeburg, Germany; olof.jannasch@klinikum-magdeburg.de; 2 Institute for Quality Assurance in Operative Medicine Ltd., Otto-von-Guericke-University Magdeburg, 39106 Magdeburg, Germany; martin.schwanz@googlemail.com (M.S.); ronny.otto@med.ovgu.de (R.O.); hans.lippert@med.ovgu.de (H.L.); 3 Department for General and Colorectal Surgery, Medical University Lodz, 91-419 Lodz, Poland; michal.mik@umed.lodz.pl; 4 Department for Surgery, University Hospital Knappschaftskrankenhaus, Ruhr-University Bochum, In der Schornau 23-25, 44892 Bochum, Germany

**Keywords:** gastric cancer, surgery, lymphadenectomy, lymph node ratio, survival

## Abstract

Background: Lymphadenectomy is a fundamental part of surgical strategy in patients with gastric cancer. Lymph node (LN) status is a key point in assessment of prognosis in gastric cancer. The LN ratio (LNR)—number of positive LNs/number of sampled LNs—offers a new approach for predicting survival. The aim of the study was to find factors affecting LN yield and the impact of LNR on 5-year survival. Methods: Prospective multicenter quality assurance study. Only LN-positive patients were included in the LNR calculations. Results: 4946 patients from 149 hospitals were enrolled. The inclusion criteria were met by 1884 patients. Patients were divided into two groups: Group 1 (<16 LN), 456 patients and Group 2 (≥16 LN), 1428 patients. The multivariate analysis found G2 (OR 1.98; 95%CI 1.11–3.54), G3 (OR 2.15; 95%CI 1.212–3.829), UICC-stage II (OR 1.44; 95%CI 1.01–2.06) and III (OR 1.71; 95%CI 1.14–2.57), age < 70 (OR 1.818 95%CI 1.19–2.78) and female gender (OR 1.37; 95%CI 1.00–1.86) as independent factors of ≥16 LN yield. Patients with a LNR ≥ 0.4 have a lower probability of survival (*p* = 0.039 and <0.001) than patients with a LNR = 0.1. Patients with UICC-II have a lower probability of survival than UICC-I (*p* = 0.023). Age 70–80 (*p* = 0.045) and > 80 years (*p* = 0.003) were negative prognostic factors for long-term survival. Conclusion: Long-term survival is directly related to adequate lymphadenectomy. LNR could be superior to pN-stage for estimating survival and adds remarkable nuances in prognosis compared to UICC-stage. LNR also appears valid, even in the case of insufficient LN yield.

## Simple Summary

Long-term survival of patients with gastric cancer is still poor. Adequate lymphadenectomy is one of the key factors that influence the long-term results of surgery. However, there is still no complete knowledge about factors influencing the lymph node yield. In our study, we analyzed these factors. For many years, the TNM system has allowed estimating long-term survival, but there are many indications that the system, along with UICC/AJCC, should be enriched with additional elements. Lymph node ratio (LNR) seems to be such an element. In this study, we performed a comprehensive analysis of the effect of LNR on long-term survival in patients with gastric cancer.

## 1. Introduction

One of the most common forms of cancer is gastric carcinoma, with an estimated 1,033,701 new cases being reported in 2018, ranking it No. 6 of all cancers. It is also unfortunately associated with a high mortality rate: a total of 782,685 deaths reported, representing the second leading cause of cancer-related death worldwide [1]. The highest incidence was noted in East Asia, at 35.4 per 100,000 in men, compared to Western Europe, at 8.8 and 3.3 in Western Africa [2]. It is characterized by a relative 5-year survival of 33% in women and 31% in men and relative 10-year survival of 30% in women and 28% in men. The median age of diagnosis in Germany in 2018 was 76 years for women and 71 years for men [3].

A fundamental part of surgical strategy is lymphadenectomy. Regional lymph nodes (LN) can be assigned to compartment I (No. 1–6 along the small and large gastric curvature) and compartment II (No. 7–12 and 14). In carcinomas of the cardia, LN located paraesophageally in the lower mediastinum, in the hiatus and infra- and supradiaphragmatically, are assigned to compartment III (No. 19, 20, 110 and 111) [4]. D1-lymphadenectomy is understood as a dissection of the perigastric LN 1–6 (compartment I), while D2-lymphadenectomy corresponds to an additional dissection of the LN 7–11 (compartment II) along the large arteries of the stomach. An additional lymphadenectomy is designated as D3 (Table 1).

Some also suggest D2-plus lymphadenectomy (removal of the “posterior” lymph node stations located behind the hepatic artery (no. 8p), the hepatoduodenal ligament (no. 12b/p), the retropancreatic nodes (no. 13), and the para-aortic area (no. 16a2/b1)) with low morbidity and mortality rates and with benefits in survival [4].

A minimum of 25 regional LN should be removed and examined histopathologically. A classification of pN0 is only possible after the excision and histological examination of at least 16 regional LN [5,6,7]. A curative treatment for gastric carcinoma includes a R0-resection with a D2-lymphadenectomy; however, in selected patients, when necessary, D2-plus or D3 lymphadenectomy should also be considered [4,8].

Survival is determined by patient age, depth of invasion, LN involvement, lymph node ratio (LNR), lymphatic vessel invasion, tumor size, type of surgery, Lauren-classification, and location of infiltrated LN [9]. Early results depend on postoperative course, and anastomotic leak is one of the most important causes of postoperative morbidity and mortality [10]. The 5-year survival rate is estimated to be around 50.4%. However, more specifically, it has been found to be 89.3% for pT1, 72.4% pT2, 36.9% pT3 and 23.7% pT4; for pN0 75.2%, pN1 68.8% pN2 46.7% pN3 and 21.3% [11]; and 93.2% for UICC-stage I, 72.4% stage II, 39.1% stage III and 5.2% stage IV [12].

LN status seems to be a key point in assessment of prognosis in gastric cancer, although the best method to assess LN involvement remains debatable [13]. The LNR, defined as number of positive LNs divided by the number of sampled LNs, offers a new approach for predicting survival, with higher LNR significantly correlating with lower 5-year survival [14]. LNR-based methods allow more accurate estimation of survival compared to TNM classification alone [15,16]. Indeed, its prognostic importance has already been confirmed for colon and pancreatic cancer [17,18].

## 2. Materials and Methods

### 2.1. Patients and Data Collection

Data were collected as part of a prospective multicenter quality assurance study (Institute for Quality Assurance in Operational Medicine at the Otto von Guericke University, Magdeburg, Germany); however, taking into account the limitations of our study, we regard the outcomes as preliminary results. P, which have to be confirmed by further research.

Participation was voluntary, and all participants gave their written informed consent to take part. Considering the character of the study design (observational prospective study for quality control), participating hospitals included three levels of care (regional, institute, university).

According to the ethics committee of Otto-von-Guericke University, Magdeburg, no ethical votum was required due to the observational character of the study. All centers were sending their data to the same database form (Quality Assurance Study Protocol Database) built for the study.

The following inclusion criteria were selected:

- Histologically confirmed primary gastric adenocarcinoma;
- Documented lymphadenectomy;
- R0-resection.

Exclusion criteria comprised:

- UICC stage 0;
- UICC stage IV;
- Pre-existing infiltration of neighboring organs (invasion of the duodenum or esophagus alone did not lead to exclusion);
- Postoperative mortality.

Only lymph node-positive patients were included in the LNR calculations. The cohort was then divided into two groups based on German guidelines specifying that an excision of at least 16 LN is necessary for an assignment “pN0” [19].

Based on the available literature, four cut-off values were chosen: 0.1 (0.01–0.1), 0.2 (0.11–0.02), 0.4 (0.21–0.4) and >0.4 [14,20,21,22,23,24].

### 2.2. Statistical Analysis

All data were integrated into Access databases after being checked for plausibility and completeness. The analysis was performed with IBM^®^ SPSS^®^ Statistics, version 24.0.0; copyright 1989–2016, SPSS Inc. (Chicago, IL, USA).

Continuous variables were described by mean, standard deviation, minimum, lower quartile, median, upper quartile and maximum. Categorical variables were represented by their absolute and relative frequencies and were compared using the chi-square test. To reject the null hypothesis, a *p*-value < 0.05 was assumed to be statistically significant.

For individual frequencies below 5 and a four-field table, Fisher’s exact test was used. Systematic differences between two groups, such as BMI, sex or ASA-classification, were examined with the t-test if the variables were normally distributed and with the Mann–Whitney U-test if they were not.

Categorical variables with more than two values were compared using analysis of variance, for parametric data, or the Kruskal–Wallis test, for non-parametric data. For normal distribution, the Shapiro–Wilk test was used.

Multivariate logistic regression analysis was performed to predict the relationships between various independent variables and lymph node yield, as the dependent discrete variable. The non-parametric estimates of survival were presented using the distribution-independent Kaplan–Meier curve. All patients who had not died before October 2016 were included in the current survival determination.

Differences were compared in terms of survival using the log-rank test. Median survival and the associated 95% confidence interval were calculated. Survival data were analyzed using Cox regression.

## 3. Results

From January 2007 to December 2012, 4946 patients from 149 hospitals were enrolled and were followed up until October 2016. The reasons for exclusion: UICC 0, IV: 943 patients; cancer without histological confirmation: 418 patients; invasion of neighboring organs: 489 patients; mortality: 216 patients; resection R1: 372 patients; resection R2: 311 patients; incomplete data: 313 patients. The inclusion criteria were met by 1884 patients. Participating departments reported, on average, 33 (1–217) patients. The median follow-up was 61 months. Of the 1884 patients included, 1118 gave their consent to follow-up; follow-up data were available for 975 patients (87.2%).

A mean of 24.4 ± 0.65 LN were resected and 2.6 ± 0.25 were positive. The mean LNR was 0.1068. The patients were divided into two groups for further analysis: Group 1 (<16 LN) comprised 456 patients and Group 2 (16 or more LN) 1428 patients.

### 3.1. Lymph Node Yield Analysis

A total of 700 women and 1180 men were examined. The mean age was 69.3 (±1.0) years in Group 1 and 66.9 (±0.6) in Group 2 (*p* < 0.001). Mean BMI was 26.4 and did not differ between groups. The most common procedure was total (35.1%) or subtotal (25.9%) gastrectomy, performed in 61% of cases, followed by cardiac resection (7.9%), distal gastrectomy with gastrojejunostomy (7.7%) and transhiatal extended gastrectomy (6.6%). The highest numbers of LN were obtained in transhiatal extended gastrectomy. Systematic lymphadenectomy resulted in a significantly higher lymph node yield (*p* < 0.001) compared with limited resection. On average, one lymph node was affected in Group 1, and more than three in Group 2 (*p* < 0.001). Lymphatic invasion (L1) could be detected in 786 (41.9%) specimens (Group 1 n = 160, Group 2 n = 626, *p* < 0.001). Venous invasion (V1) could be detected in 212 (12.5%) patients (Group 1 n = 46, Group 2 n = 166; *p* = 0.737). Information on the histological tissue of the carcinomas was available for 1361 patients. Gastric adenocarcinoma was found in almost 90% of the patients (n = 1236). The tubular and signet ring cell carcinomas accounted for the largest share, with a total of 68%, followed by mucinous and papillary adenocarcinomas. Significantly fewer lymph nodes were removed from mucinous adenocarcinomas (*p* = 0.038), significantly more from signet ring cell carcinomas (*p* = 0.026). Total (52.9%) or subtotal (28.4%) gastrectomy was performed in 81.3% of all patients, followed by proximal resection (3.1%). Total gastrectomy and transhiatal extended gastrectomy were the most successful in terms of lymph node yield. Details are given in Table 2.

The univariate analysis found the following factors to affect LN yield ≥ 16 LN: neoadjuvant treatment, grading, pT-stage, pN-stage, lymphatic invasion, venous invasion, UICC-stage, Lauren-classification, and localization at the gastroesophageal junction (*p* < 0.001) and the antral/pyloric region (*p* = 0.030) (Table 3).

The multivariate logistic regression analysis found grading (grade 2 and 3), UICC-stage, age < 70 years and sex to be independent factors influencing excision of ≥16 LN (Table 4).

### 3.2. Long-Term Survival Analyses

**Figure 1:**
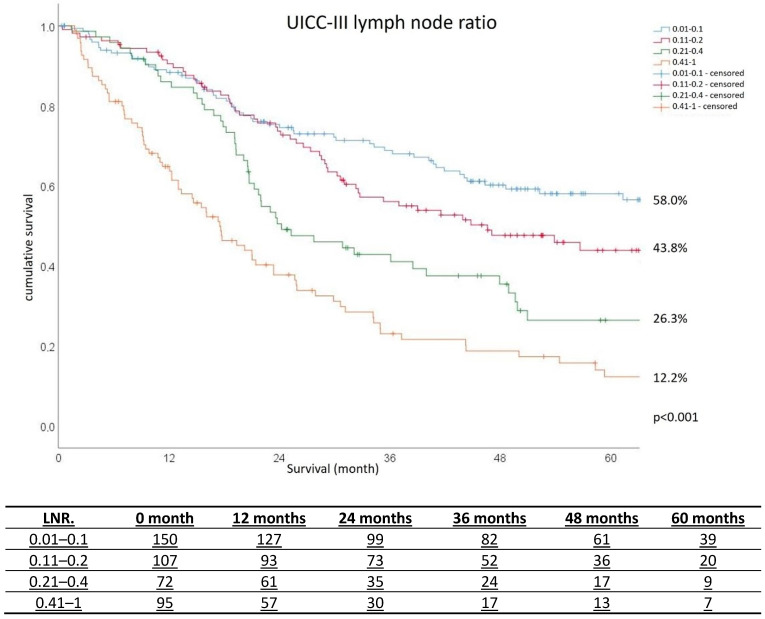
Five-year survival as a function of lymph node ratio.

Age, pT-stage, UICC-stage and LNR were significant prognostic factors for survival (*p* < 0.001). Postoperative survival was found to decrease with increasing age. Median survival was 65.2 months (<70 year group), 54.7 months (70–80 years) and 44.5 months (>80 years) (*p* < 0.001). Survival also decreased with increasing pT-stage. Overall survival was 95.0 months, but only 56.7 months for patients with pT3 and 103.4 months for pT2. No information for stage pT1 can be given, as the probability of survival at the end of the follow-up was over 50%. Five-year survival also decreased with increasing UICC-stage: 75.2% for UICC-I, 65.6% for UICC-II and 30.8% for UICC-III. It also correlated significantly with LNR (*p* < 0.001), even in the first few months: 5-year survival in UICC-III was found to be 58.0% for LNR 0.1, 43.8% for LNR 0.2, 26.3% for LNR 0.4 and 12.2% for LNR > 0.4 (Figure 1).

The multivariate analysis found patients with a LNR of 0.4 and > 0.4 have a lower probability of survival (*p* = 0.039 and <0.001) compared to patients with a LNR of 0.1. Furthermore, patients with UICC-II gastric cancer have a lower probability of survival than patients with UICC-I (*p* = 0.023). Cox regression also identified age 70–80 years (*p* = 0.045) and over 80 years (*p* = 0.003) as negative prognostic factors for long-term survival (Table 5).

## 4. Discussion

This study included one of the largest cohorts in any study about LNR to date. The findings indicate that stratification with a 4 LNR cut-off was statistically valid. LNR allows better differentiation and more precise prediction of outcomes among LN-positive gastric carcinomas.

### 4.1. Lymph Node Yield

A strong positive correlation was observed between the number of LNs removed and the number of LNs affected (*p* < 0.001). Similar results were obtained by Huang, who reported a mean number of 23.1 ± 8.6 LN removed per patient [25], and in another Chinese study with 1470 patients, where a mean of 25.8 ± 12.8 LN were removed [26]. Even in patients with LN-negative gastric cancer, survival improved when increased numbers of LN were removed [26]. In a Korean study [27], patients with pT1 tumor, pN0-status and UICC-1 stage demonstrated a significantly worse prognosis when fewer than 16 LN were removed compared to those with 16 or more.

The highest numbers of LN were removed in G3 carcinomas, whereas the LN yield in G1 and G4 was particularly poor. Similar results were obtained in a study from Beijing [28], where most patients were found to have G3 (48.2%) and G2 (22.1%); however, G4 carcinomas were more common (26.2%) than in the present study (2.2%), probably mostly due to our exclusion criteria. Grading was also found to be a significant prognostic factor for LN yield in a univariate analysis from Finland [29]. G4 tumors are often marked by fast growth and early tumor spread; as such, in some cases, intraoperative findings may lead to limited resection, resulting in reduced numbers of removed LN. We cannot find the scientific explanation of relations between lymph node yield, grading, venous and lymphatic invasion found in our study; it probably would need more specified research.

UICC-stage is determined by depth of invasion, LN involvement and metastasis. Interestingly, regarding depth of invasion, the most common classification in the present study was pT3, at 46.1% of examined specimens; this was also found to be the most common form in a study by Chen, at 40.8% [27], and a US study, at 36.4% [23]. Our data indicate that the number of LNs removed increased with the depth of invasion. We would hypothesize that size of tumor is correlated with the depth of invasion (pT stage) [11] and thorough this influences the decision to perform, and the extent of, fatty tissue dissection.

The mean age of patients in this study was 66.7 years for men and 69.2 years for women. These were slightly lower than the mean values in the German database from the Robert Koch Institute, listing all gastric cancer patients (71 years for men and 76 years for women) [3]. Age group clearly affected the extent of LN yield, with age 70–80 years having the most LN removed. This was also confirmed in a Chinese study, which found older patients to have more advanced and larger tumors [30]. This might explain the difficulties associated with lymphadenectomy in the elderly. Also, older patients have more comorbidities that might require more limited surgery, i.e., shorter operations with a lower risk of complications. Mayol-Oltra et al. [31] report that the presence of comorbidities in older patients leads to fewer LN being removed. This might be the reason for fewer LN being removed in patients aged > 80 years in our study.

Fewer LNs tend to be removed from male patients. This may be significant, as gastric carcinoma is more common in men. The sex ratio in the present study was 1:1.69 women to men. A similar ratio, i.e., 1:1.65, was noted for 2018 German data from the Robert Koch Institute [3]; however, a study by the Korean Cancer Association found the ratio to fall from 1:1.8 in 2004 to 1:1.5 in 2014 [32]. The numbers of new cases of gastric carcinoma seem to approximate between the sexes.

Neoadjuvant treatment led to a significant increase in harvested LN (*p* = 0.009). In contrast, Li et al. [33] report that preoperative chemoradiotherapy caused a decrease in LN yield (25.5). Chemotherapy alone (31.0) also resulted in a decreased LN yield compared to patients not receiving neoadjuvant treatment (32.0). The extent to which neoadjuvant radiotherapy affects LN excision remains to be investigated, but it has been demonstrated that preoperative radiotherapy can increase the chance of 5-year survival from 19.75% to 30.10% and 10-year survival from 13.30% to 20.26% (*p* = 0.0094). Radiotherapy also reduced the rate of LN metastases from 84.9% to 64.3% [34]. Other studies [20,35] using neoadjuvant chemotherapy demonstrated an increase in free resection margin and a decrease in the number of local LN metastases.

In recent years, there has been a significant shift towards immunotherapy as a promising avenue for the treatment of advanced malignancies. Clinical trials have also demonstrated the efficacy of neoadjuvant immunochemotherapy in patients with locally advanced gastric cancer [36,37]. Zhou P. et al. [38] found that high LNR (≥33%) was an independent prognostic factor for overall survival (HR 6.258, 95% CI 1.798–21.778; *p* = 0.004) and progression-free survival (HR 3.431, 95% CI 1.341–8.780; *p* = 0.010).

Unfortunately, detailed information about neoadjuvant treatment was not included in the protocol and thus was not assessed in the present study. We realize that it could be one of the limitations of our study.

The gastric carcinomas demonstrated similar localizations, as noted in Chinese studies by Chen et al. [28] and Zhao et al. [39]. Most carcinomas were found in the lower third (antrum/pylorus) followed by the middle third (corpus). Our data indicate that tumor location and LN yield are significantly related. Recent studies indicate that tumors may also occur more frequently in the upper third: one Turkish study [40] found cardia carcinomas to be more common than those in other parts (*p* = 0.004).

### 4.2. Lymph Node Ratio

The univariate and multivariate analyses found age group, depth of invasion, UICC-stage and LNR to be independent prognostic factors for long-term survival. This has been confirmed in other studies [39,41,42]. LNR and intestinal histological type were found to be independent prognostic factors in a Japanese study [20], and pT-stage, pN-stage and extent of surgery in a Polish study [22]. Son et al. found age ≥ 60 years, male sex, pT-stage, pN-stage, insufficient number of examined LN and upper tumor localization to be significant risk factors for survival [27], while age, UICC-stage, resection margin and LNR were indicated as independent prognostic factors in a study from the NYU School of Medicine [21].

While depth of invasion is a component of UICC-stage and can independently predict survival, LNR seems to be better suited to predict survival than pN-stage. LNR appears to effectively predict 5-year survival regardless of cut-off value. It was found to be 63.4% at a cut-off of LNR 0.15, 46.9% at LNR 0.4, and 22.6% at LNR of 0.41–1 in a Chinese study [39]. A meta-analysis of 27 articles comprising 11,441 patients with gastric cancer and radical surgery found higher LNR to be clearly associated with shorter overall survival; however, the studies displayed high heterogeneity [43].

In the present study, the UICC-III patients demonstrated 5-year survival values of 58.0% (LNR 0.01–1), 43.8% (0.11–0.2), 26.3% (0.21–0.4) and 12.2% (0.41–1). LNR allows a more accurate and detailed prognosis estimation for pN-positive gastric cancer patients compared to AJCC/TNM-staging alone [44,45]. In a population-based study by Huang et al. [46], a total of 13,027 patients with IIIA category (8th AJCC) were classified into subgroups rIIB, rIIIA, rIIIB and rIIIC with the help of LNR; the patients demonstrated a similar 5-year overall survival rate to our present cohort, i.e., from 66.7% to 5.1%. Combined TNM and LNR seem also more reliable for prognosis in patients with neoadjuvant treatment compared to TNM-classification alone, as shown in a large population-based study from Chen et al. [16] with 1791 patients.

The distribution of LN involvement in this study roughly corresponds to that observed in an US review comprising 9357 patients. In both cases, the largest group included patients without local LN metastases: 45.1% in the US study compared to 55.5% in this study. Individual pN1–pN3 stages differed by only a few percent between studies (pN1: 19.5% vs. 17.0%; pN2: 16.9% vs. 13.6%; pN3: 18.5% vs. 13.8%) with slightly higher values noted in all groups in the American study [23]. A Chinese study [41] (935 patients) comparing three different LN staging systems in survival prognosis following D2 lymphadenectomy in gastric cancer found LNR to be superior to pN-stage.

Our data indicate a significant positive relationship between the number of positive LNs and the numbers of LN removed (*p* < 0.001). Similar results were found in a Chinese study by Zhao [39], in which 858 patients were classified as free of metastases (pN0), 511 were assigned to pN1, 494 to pN2 and 712 to pN3. It is important to note that care should be taken to completely remove the individual LN compartments; this is particularly important for carcinomas with a low UICC-stage. It was also found that removing higher numbers of LN was also related to improved overall survival [47].

Our data indicate that LNR was a significant prognostic factor for long-term survival. This has also been confirmed in several other studies [14,20,22,23,39,41,42,45,46,48] using a variety of cut-off points ranging from LNR 0 to 0.8. The numbers of patients in these studies range from 73 to 9357. Our classification with LNR cut-off values of 0.1, 0.2, 0.4 and >0.4 was found to give a precise survival prognosis for patients with 16 or more LN removed and who are lymph node-positive.

Interestingly, retrospective data from Sun Yat-sen University Hospital in China [46] (2205 patients) displayed no difference in survival prognosis for patients with ≥16 LN removed (C-index: 0.77) compared to patients with ≤15 LN removed (C-index: 0.75). Also, among patients with ≥UICC-2 stage, Son et al. [27] found no difference in overall survival between patients with <16 removed LN and those with 16 or more. As such, it may be that LNR could be a useful indicator even in the event of insufficient LN harvest.

### 4.3. Limitations of the Study

We realize that our study has several limitations. Participating hospitals were not obliged to follow any specific pathological and surgical protocol. The decisions (surgical and pathological) were made only according to current guidelines. We realize that the lack of a uniform protocol might have influenced our results. In the database protocol, the only ASA classification was included to describe patient general stage and, indirectly, comorbidities. Information on particular comorbidities was not collected. The obtained data concerned the neoadjuvant therapy without information on the details of the therapy (type of treatment, time of duration). For that reason, we were not able to provide the precise impact of individual elements of neoadjuvant therapy but only its overall impact on lymph node yield.

## 5. Conclusions

Long-term survival of patients with gastric carcinoma is directly related to adequate lymphadenectomy. LNR is superior to pN-stage for estimating survival and adds remarkable nuances in prognosis compared to UICC-stage. LNR also appears valid even in the case of insufficient LN yield. We hence recommend that LNR should be incorporated into staging systems (UICC/AJCC) and into the decision process for adjuvant strategies.

## Figures and Tables

**Table 1:** The extension of lymphadenectomy in gastric cancer.

	Numbers of Lymph Node Stations		
Type of Lymphadenectomy	Compartment I	Compartment II	Compartment III
D1	1, 2, 3, 4, 5, 6	-	-
D2	1, 2, 3, 4, 5, 6	7, 8, 9, 10,11	-
D2-plus	1, 2, 3, 4, 5, 6	7, 8, 9, 10,11	12, 13, 16
D3	1, 2, 3, 4, 5, 6	7, 8, 9, 10,11	12, 13, 14, 15, 16

Lymph node stations: 1: right cardiac nodes, 2: left cardiac nodes, 3: nodes along lesser curvature, 4: nodes along greater curvature, 5: suprapyloric nodes, 6: infrapyloric nodes, 7: nodes along left gastric artery, 8: nodes along common hepatic artery, 9: nodes along celiac trunk, 10: nodes at splenic hilum, 11: nodes along splenic artery, 12: nodes at the hepatoduodenal ligament, 13: retropancreatic nodes, 14: nodes at the root of mesentery, 15: nodes along the middle colic vein, 16: para-aortic nodes.

**Table 2:** Effect of patient and tumor-related factors on lymph node yield.

		Group 1 (n/%)	Group 2 (n/%)	*p*-Value *
Sex	Male	213/68.6	868/60.9	<0.001
	Female	143/31.4	557/39.1	
ASA-classification	ASA I	23/5.1	121/8.6	<0.001
	ASA II	199/44.3	767/54.5	
	ASA III	214/47.7	495/35.2	
	ASA IV	13/2.9	24/1.7	
Grading	G1	39/8.6	59/4.1	<0.001
	G2	159/34.9	429/30.2	
	G3	228/50.1	836/58.8	
	G4	14/3.1	27/1.9	
Histology	Papillary adenocarcinoma	30/6.6	97/6.8	0.874
	Tubular adenocarcinoma	114/25.0	371/26.0	0.677
	Mucinous adenocarcinoma	56/12.3	128/9.0	0.038
	Signet ring cell carcinoma	89/19.5	351/24.6	0.026
	Undifferentiated carcinoma	25/5.5	84/5.9	0.750
	Small cell carcinoma	2/0.4	0/0.0	-
	Squamous cell carcinoma	4/0.9	7/0.5	0.345
	Adenosquamous carcinoma	0/0.0	3/0.2	0.327
Surgical approach	Laparotomy	430/95.2	1411/99.1	<0.001
	Laparoscopic	18/4.0	7/0.5	
Surgical procedure	Proximal resection	36/8.2	22/1.5	<0.001
	Subtotal/distal gastrectomy	166/37.9	364/25.6	0.112
	Total gastrectomy	160/36,4	827/58.1	<0.001
	Transhiatal extended gastrectomy	30/6.8	144/10.1	0.024
	Transthoracic extended gastrectomy	17/3.9	33/2.3	0.101
	Thoraco-abdominal esophagogastrectomy	16/3.6	23/1.6	0.013
	Other gastrectomy	14/3.2	12/0.8	<0.001
Neoadjuvant treatment	No	363/80.3	1054/74.3	0.009
	Yes	89/19.7	365/25.7	
Localization	Gastroesophageal junction	125/27.4	280/19.6	<0.001
	Fundus	19/4.2	45/3.2	0.297
	Corpus	142/31.1	553/38.7	0.003
	Antral/pyloric region	181/39.7	601/41.5	0.366
Invasion depth	pT0	0/0.0	2/0.1	<0.001
	pT1	200/44.0	460/32.3	
	pT2	88/25.1	262/18.4	
	pT3	167/36.7	699/49.1	
pN-stage	pN0	288/63.6	756/53.0	<0.001
	pN1	81/17.9	239/16.8	
	pN2	52/11.5	203/14.2	
	pN3	32/7.1	228/16.0	
UICC-stage	I	249/54.6	578/40.5	<0.001
	II	117/25.7	427/30.6	
	III	90/19.7	413/28.9	

* Chi^2^-Test.

**Table 3:** Univariate analysis. Prognostic factors predicting lymph node yield ≥ 16.

		LK-Quotient (Mean ± SD)	*p*-Value
Sex	Male	0.11 ± 0.19	0.132 **
	Female	0.10 ± 019	
Age groups <70, 70–80, >80	<70	0.11 ± 0.19	0.213 *
	70–80	0.11 ± 0.19	
	>80	0.12 ± 0.21	
BMI	<18.5	0.15 ± 0.23	0.682 *
	18.5–24.9	0.11 ± 0.20	
	≥25	0.10 ± 0.18	
Lauren classification	None	0.11 ± 0.18	<0.001 *
	Intestinal	0.08 ± 0.16	
	Diffuse	0.14 ± 0.23	
	Mixed	0.10 ± 0.17	
Localization	Fundus	0,12 ± 0.20	0.889 **
	Gastroesophageal junction	0.14 ± 0.20	<0.001 **
	Corpus	0.11 ± 0.20	0.923 **
	Antrum/pylorum	0.10 ± 0.20	0.030 **
ASA-classification	I	0.10 ± 0.19	0.440 *
	II	0.11 ± 0.20	
	III	0.11 ± 0.19	
	IV	0.05 ± 0.10	
Neoadjuvant treatment	**No**	0.10 ± 0.19	<0.001 **
	**Yes**	0.12 ± 0.19	
Surgical approach	Laparotomy	0.11 ± 0.19	0.548 **
	Laparoscopic	0.07 ± 0.12	
Grading	G1	0.01 ± 0.07	<0.001 *
	G2	0.08 ± 0.16	
	G3	0.13 ± 0.21	
	G4	0.11 ± 0.18	
pT-stage	pT0	0.06 ± 0.08	<0.001 *
	pT1	0.02 ± 0.08	
	pT2	0.08 ±0.15	
	pT3	0.17 ± 0.23	
pN-stage	pN0	0.01 ± 0.03	<0.001 *
	pN1	0.06 ± 0.05	
	pN2	0.15 ± 0.07	
	pN3	0.47 ± 0.22	
Lymph invasion	**L0**	0.03 ± 0.09	<0.001 **
	**L1**	0.20 ± 0.23	
Venous invasion	V0	0.09 ± 0,17	<0.001 **
	V1	0.24 ± 0.27	
UICC-stage	I	0.003 ± 0.01	<0.001 *
	II	0.05 ± 0.10	
	III	0.31 ± 0.24	

* Kruskall–Wallis Test, ** Mann–Whitney U-Test.

**Table 4:** Factors predicting lymph node yield of ≥16 LN. Results of the logistic regression.

		Odds Ratio (95% CI)	*p*-Value
Grading	G1	1	
	G2	1.982 (1.110–3.541)	**0.021**
	G3	2.154 (1.212–3.829)	**0.009**
	G4	0.739 (0.268–2.036)	0.558
UICC-stage	I	1	
	II	1.441 (1.008–2.060)	**0.045**
	III	1.707 (1.135–2.568)	**0.010**
Age groups	>80	1	
	<70	1.818 (1.188–2.783)	**0.006**
	70–80	1.358 (0.874–2.109)	0.173
Sex	Men	1	
	Women	1.365 (1.000–1.863)	**0.050**
Venous invasion	No (V0)	1	
	Yes (V1)	0.647 (0.411–1.016)	0.059

**Table 5:** Results of Cox regression analysis for 5-year survival.

		Hazard Ratio (95% CI)	*p*-Value
Lymph node ratio	0.01–0.1	1	
	0.11–0.2	1.207 (0.770–1.893)	0.413
	0.21–0.4	1.652 (1.027–2.659)	**0.039**
	0.41–1	2.746 (1.740–4.333)	**<0.001**
UICC-stage	I	1	
	II	0.485 (0.260–0.905)	**0.023**
	III	0.849 (0.436–1.654)	0.630
Age groups	<70	1	
	70–80	1.374 (1.008–1.874)	**0.045**
	>80	1.806 (1.225–2.663)	**0.003**

## Data Availability

The data supporting the findings of this study are not publicly available due to privacy concerns.

## Abbreviations

The following abbreviations are used in this manuscript:

LNR	Lymph Node Ratio
TNM	Tumor Nodes Metastasis
LN	Lymph Node
BMI	Body Mass Index
UICC	Union for International Cancer Control
AJCC	American Joint Committee on Cancer
ASA	American Society of Anesthesiology

## References

[1] Bray F., Ferlay J., Soerjomataram I., Siegel R.L., Torre L.A., Jemal A. (2018). Global cancer statistics 2018: GLOBOCAN estimates of incidence and mortality for 36 cancers in 185 countries. CA Cancer J. Clin..

[2] Torre L.A., Bray F., Siegel R.L., Ferlay J., Lortet-Tieulent J., Jemal A. (2015). Global cancer statistics, 2012. CA Cancer J. Clin..

[3] Robert-Koch-Institute (2020). Centre for Cancer Registry Data. www.rki.de/EN/Content/Health_Monitoring/Cancer_Registry/cancer_registry_node.html.

[4] Marrelli D., Piccioni S.A., Carbone L., Petrioli R., Costantini M., Malagnino V., Bagnacci G., Rizzoli G., Calomino N., Piagnerelli R. (2024). Posterior and Para-Aortic (D2plus) Lymphadenectomy after Neoadjuvant/Conversion Therapy for Locally Advanced/Oligometastatic Gastric Cancer. Cancers.

[5] Japanese Gastric Cancer Association (2023). Japanese Gastric Cancer treatment Guidelines 2021 (6th edition). Gastric Cancer.

[6] Coburn N., Cosby R., Klein L., Knight G., Mamazza J., Mercer C.D., Ringash J. (2017). Staging and surgical approaches in gastric cancer: A clinical practice guideline. Curr. Oncol..

[7] Wu W., Dong P., Wu X., Li M., Ding Q., Zhang L., Yang J., Weng H., Ding Q., Tan Z. (2014). Three-step method for systematic lymphadenectomy in gastric cancer surgery using the ‘curettage and aspiration dissection technique’ with Peng’s multifunctional operative dissector. World J. Surg. Oncol..

[8] Lordick F., Al-Batran S.E., Arnold D., Borner M., Bruns C.J., Eisterer W., Faber G., Gockel I., Köberle D., Lorenzen S. In Kooperation Mit der AIO. Onkopedia Leitlinen—Magenkarzinom 2023. www.onkopedia.com/de/onkopedia/guidelines/magenkarzinom/.

[9] Yu J., Yang D., Wei F., Sui Y., Li H., Shao F., Li Y. (2008). The staging system of metastatic lymph node ratio in gastric cancer. Hepatogastroenterology.

[10] Radulescu D., Baleanu V.D., Padureanu V., Radulescu P.M., Bordu S., Patrascu S., Socea B., Bacalbasa N., Surlin M.V., Georgescu I. (2020). Neutrophil/Lymphocyte Ratio as Predictor of Anastomotic Leak after Gastric Cancer Surgery. Diagnostics.

[11] Lu J., Huang C.M., Zheng C.H., Li P., Xie J.W., Wang J.B., Lin J.X. (2013). Consideration of tumor size improves the accuracy of TNM predictions in patients with gastric cancer after curative gastrectomy. Surg. Oncol..

[12] Wang W., Li Y.F., Sun X.W., Chen Y.B., Li W., Xu D.Z., Guan X.X., Huang C.Y., Zhan Y.Q., Zhou Z.W. (2010). Prognosis of 980 patients with gastric cancer after surgical resection. Chin. J. Cancer.

[13] Díaz Del Arco C., Ortega Medina L., Estrada Muñoz L., García Gómez de Las Heras L., Fernández Aceñero M.J. (2021). Pathologic Lymph Node Staging of Gastric Cancer. Am. J. Clin. Pathol..

[14] Lee S.R., Kim H.O., Son B.H., Shin J.H., Yoo C.H. (2012). Prognostic significance of the metastatic lymph node ratio in patients with gastric cancer. World J. Surg..

[15] A La-teng B.L., Li Y.M., Liu C.G., Wang B.B., Xu H.M., Chen J.Q., Wang S.B., Lu P. (2012). Prognostic value of metastatic lymph node ratio in gastric cancer. Chin. J. Gastrointest. Surg..

[16] Chen J.X., Sun J.W., Wang Y., Pan T., Zhuang L.P., Lin L.Z., Lv B.C. (2022). Lymph node ratio-based the ypTNrM staging system for gastric cancer after neoadjuvant therapy: A large population-based study. Surg. Today.

[17] Karaca C.A., Coker A. (2019). Prognostic Value of Metastatic Lymph Node Ratio in Pancreatic Cancer. Indian J. Surg. Oncol..

[18] Mroczkowski P., Kim Samuel Otto R., Lippert H., Zajdel R., Zajdel K., Merecz-Sadowska A. (2024). Prognostic value of metastatic lymph node ratio and identification of factors influencing the lymph node yield in patients undergoing curative colon cancer resection. Cancers.

[19] German Cancer Society, German Cancer Aid, AWMF Guideline Programme on Oncology: S3-Leitlinie Diagnostik und Therapie der Adenokarzinome des Magens und ösophagogastralen Übergangs (Version 2, 2019); AWMF Register Number: 032/009OL. www.leitlinienprogramm-onkologie.de/leitlinien/magenkarzinom/.

[20] Ema A., Yamashita K., Sakuramoto S., Wang G., Mieno H., Nemoto M., Shibata T., Katada N., Kikuchi S., Watanabe M. (2014). Lymph node ratio is a critical prognostic predictor in gastric cancer treated with S-1 chemotherapy. Gastric Cancer.

[21] Melis M., Masi A., Pinna A., Cohen S., Hatzaras I., Berman R., Pachter L.H., Newman E. (2015). Does lymph node ratio affect prognosis in gastroesophageal cancer?. Am. J. Surg..

[22] Spychała A., Nowaczyk P., Murawa D. (2015). Comparison of Lymphatic System Staging Classifications in Patients with Gastric Cancer. Pol. Przegl Chir..

[23] Kutlu O.C., Watchell M., Dissanaike S. (2015). Metastatic lymph node ratio successfully predicts prognosis in western gastric cancer patients. Surg. Oncol..

[24] Wu X.J., Miao R.L., Li Z.Y., Bu Z.D., Zhang L.H., Wu A.H., Zong X.L., Li S.X., Shan F., Ji X. (2015). Prognostic value of metastatic lymph node ratio as an additional tool to the TNM stage system in gastric cancer. Eur. J. Surg. Oncol..

[25] Huang C.M., Lin J.X., Zheng C.H., Li P., Xie J.W., Lin B.J., Wang J.B. (2010). Prognostic impact of metastatic lymph node ratio on gastric cancer after curative distal gastrectomy. World J. Gastroenterol..

[26] Jiao X.G., Deng J.Y., Zhang R.P., Wu L.L., Wang L., Liu H.G., Hao X.S., Liang H. (2014). Prognostic value of number of examined lymph nodes in patients with node-negative gastric cancer. World J. Gastroenterol..

[27] Son T., Hyung W.J., Lee J.H., Kim Y.M., Kim H.I., An J.Y., Cheong J.H., Noh S.H. (2012). Clinical implication of an insufficient number of examined lymph nodes after curative resection for gastric cancer. Cancer.

[28] Chen C.Q., Wu X.J., Yu Z., Bu Z.D., Zuo K.Q., Li Z.Y., Ji J.F. (2013). Prognosis of patients with gastric cancer and solitary lymph node metastasis. World J. Gastroenterol..

[29] Setälä L.P., Kosma V.M., Marin S., Lipponen P.K., Eskelinen M.J., Syrjänen K.J., Alhava E.M. (1996). Prognostic factors in gastric cancer: The value of vascular invasion, mitotic rate and lymphoplasmacytic infiltration. Br. J. Cancer.

[30] Liang Y.X., Deng J.Y., Guo H.H., Ding X.W., Wang X.N., Wang B.G., Zhang L., Liang H. (2013). Characteristics and prognosis of gastric cancer in patients aged ≥ 70 years. World J. Gastroenterol..

[31] Mayol-Oltra A., Marti-Obiol R., López-Mozos F., Báguena-Requena G., Ortega-Serrano J. (2013). The influence of advanced age on the morbi-mortality of gastric cancer after curative surgery. Rev. Esp. Enferm. Dig..

[32] Eom B.W., Jung K.W., Won Y.J., Yang H., Kim Y.W. (2018). Trends in Gastric Cancer Incidence According to the Clinicopathological Characteristics in Korea, 1999–2014. Cancer Res. Treat..

[33] Li Z., Li S., Bu Z., Zhang L., Wu X., Shan F., Jia Y., Ji X., Ji J. (2018). The effect of preoperative treatments on lymph node counts after total gastrectomy in esophagogastric adenocarcinoma. J. Surg. Oncol..

[34] Zhang Z.X., Gu X.Z., Yin W.B., Huang G.J., Zhang D.W., Zhang R.G. (1998). Randomized clinical trial on the combination of preoperative irradiation and surgery in the treatment of adenocarcinoma of gastric cardia (AGC)-report on 370 patients. Int. J. Radiat. Oncol. Biol. Phys..

[35] Tsuburaya A., Mizusawa J., Tanaka Y., Fukushima N., Nashimoto A., Sasako M., Stomach Cancer Study Group of the Japan Clinical Oncology Group (2014). Neoadjuvant chemotherapy with S-1 and cisplatin followed by D2 gastrectomy with para-aortic lymph node dissection for gastric cancer with extensive lymph node metastasis. Br. J. Surg..

[36] Shitara K., Rha S.Y., Wyrwicz L.S., Oshima T., Karaseva N., Osipov M., Yasui H., Yabusaki H., Afanasyev S., Park Y.K. (2024). Neoadjuvant and adjuvant pembrolizumab plus chemotherapy in locally advanced gastric or gastro-oesophageal cancer (KEYNOTE-585): An interim analysis of the multicentre, double-blind, randomised phase 3 study. Lancet Oncol..

[37] Sun X., Lyu J., Yang M., Wu K., Liu K., Li A., Shuai X., Cai K., Wang Z., Wang G. (2024). Two-Year Outcomes and Biomarker Analysis of Locally Advanced Gastric and Gastroesophageal Junction Adenocarcinoma After Neoadjuvant Chemotherapy and Immunotherapy from the Phase II WuhanUHGI001 Trial. Ann. Surg. Oncol..

[38] Zhou P., Sun X., Zeng L., Zeng X., Xie G., Liu X., Tao K., Zhang P. (2024). Lymph node ratio is a prognostic indicator for locally advanced gastric cancer after neoadjuvant immunochemotherapy. BMC Gastroenterol..

[39] Zhao L.Y., Li C.C., Jia L.Y., Chen X.L., Zhang W.H., Chen X.Z., Yang K., Liu K., Wang Y.G., Xue L. (2016). Superiority of lymph node ratio-based staging system for prognostic prediction in 2575 patients with gastric cancer: Validation analysis in a large single center. Oncotarget.

[40] Tural D., Selçukbiricik F., Akar E., Serdengeçti S., Büyükünal E. (2013). Gastric cancer: A case study in Turkey. J. Cancer Res. Ther..

[41] Wong J., Rahman S., Saeed N., Lin H.Y., Almhanna K., Shridhar R., Hoffe S., Meredith K.L. (2013). Prognostic impact of lymph node retrieval and ratio in gastric cancer: A U.S. single center experience. J. Gastrointest. Surg..

[42] Chen J.-H., Cai S.-R., Wu H., Chen S.-L., Xu J.-B., Zhai E.-T., Chen C.-Q., He Y.-L. (2016). Prognostic value of three different lymph node staging systems in the survival of patients with gastric cancer following D2 lymphadenectomy. Tumour Biol..

[43] Zhu J., Xue Z., Zhang S., Guo X., Zhai L., Shang S., Zhang Y., Lu H. (2018). Integrated analysis of the prognostic role of the lymph node ratio in node-positive gastric cancer: A meta-analysis. Int. J. Surg..

[44] Chen S., Rao H., Liu J., Geng Q., Guo J., Kong P., Li S., Liu X., Sun X., Zhan Y. (2017). Lymph nodes ratio based nomogram predicts survival of resectable gastric cancer regardless of number of examined lymph nodes. Oncotarget.

[45] Topcu R., Şahiner I.T., Kendirci M., Erkent M., Sezikli I., Tutan M.B. (2022). Does lymph node ratio (metastasis/total lymph node count) affect survival and prognosis in gastric cancer?. Saudi Med. J..

[46] Huang Z., Chen Y., Zhang W., Liu H., Wang Z., Zhang Y. (2020). Modified Gastric Cancer AJCC Staging with a Classification Based on the Ratio of Regional Lymph Node Involvement: A Population-Based Cohort Study. Ann. Surg. Oncol..

[47] Zhang Y., Tian S. (2013). Does D2 plus para-aortic nodal dissection surgery offer a better survival outcome compared to D2 surgery only for gastric cancer consistently? A definite result based on a hospital population of nearly two decades. Scand. J. Surg..

[48] Deng J., Zhang R., Wu L., Zhang L., Wang X., Liu Y., Hao X., Liang H. (2015). Superiority of the ratio between negative and positive lymph nodes for predicting the prognosis for patients with gastric cancer. Ann. Surg. Oncol..

